# Succinate Dehydrogenase and Human Disease: Novel Insights into a Well-Known Enzyme

**DOI:** 10.3390/biomedicines12092050

**Published:** 2024-09-09

**Authors:** María J. Esteban-Amo, Patricia Jiménez-Cuadrado, Pablo Serrano-Lorenzo, Miguel Á. de la Fuente, María Simarro

**Affiliations:** 1 Department of Cell Biology, Genetics, Histology and Pharmacology, Faculty of Medicine, University of Valladolid, 47005 Valladolid, Spain; mariajesus.esteban@uva.es (M.J.E.-A.); patriciajicu@gmail.com (P.J.-C.); miguelafuente@gmail.com (M.Á.d.l.F.); 2 Unit of Excellence Institute of Biomedicine and Molecular Genetics (IBGM), University of Valladolid and Spanish National Research Council (CSIC), 47003 Valladolid, Spain; 3 Mitochondrial Disorders Laboratory, Clinical Biochemistry Department, Hospital 12 de Octubre Research Institute (imas12), 28041 Madrid, Spain; pabloserralor@gmail.com; 4 Biomedical Network Research Centre on Rare Diseases (CIBERER), Instituto de Salud Carlos III, 28029 Madrid, Spain

**Keywords:** mitochondria, succinate dehydrogenase, complex II, disease

## Abstract

Succinate dehydrogenase (also known as complex II) plays a dual role in respiration by catalyzing the oxidation of succinate to fumarate in the tricarboxylic acid (TCA) cycle and transferring electrons from succinate to ubiquinone in the mitochondrial electron transport chain (ETC). Owing to the privileged position of SDH/CII, its dysfunction leads to TCA cycle arrest and altered respiration. This review aims to elucidate the widely documented profound metabolic effects of SDH/CII deficiency, along with the newly unveiled survival mechanisms in SDH/CII-deficient cells. Such an understanding reveals exploitable vulnerabilities for strategic targeting, which is crucial for the development of novel and more precise therapies for primary mitochondrial diseases, as well as for familial and sporadic cancers associated with SDH/CII mutations.

## 1. Introduction

**Figure 1:**
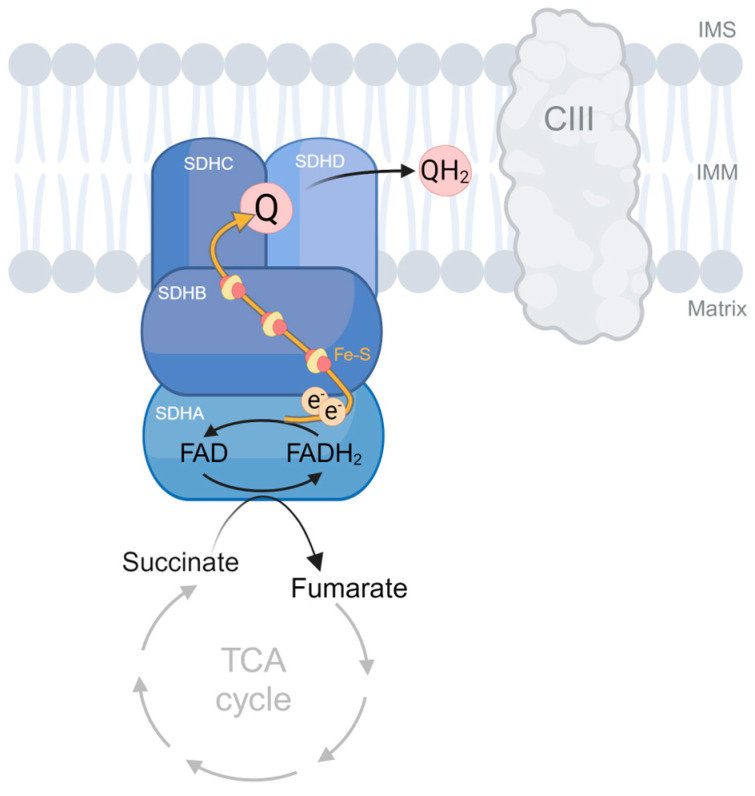
Schematic depiction of SDH/CII structure and function. The complex participates in both OXPHOS and the TCA cycle by transferring electrons from succinate to ubiquinone (Q) through its four subunits: SDHA, SDHB, SDHC, and SDHD. Fe-S, iron–sulfur clusters; Q, ubiquinone; QH2, ubiquinol; IMM, inner membrane; and IMS, intermembrane space.

OXPHOS is composed of the electron transport chain (ETC), which consists of four multimeric complexes (CI to CIV), two mobile electron carriers (ubiquinone and cytochrome c), and an ATP synthase (CV). The ETC carries out the redox reactions involved in cellular respiration while generating the proton motive force used by the CV to synthesize ATP [1]. Complex II is the smallest complex of the ETC and serves as an entry point of reducing agents into the ETC. Complex II is the only complex in which all subunits are encoded by nuclear DNA, and it is also the only complex that does not pump protons across the inner mitochondrial membrane, and so it does not contribute directly to the proton motive force. In addition to its role in OXPHOS as an ETC component, the complex also participates in the tricarboxylic acid (TCA) cycle, providing a functional link between these two essential processes. Complex II (also called succinate dehydrogenase, SDH) comprises four subunits: SDHA, SDHB, SDHC, and SDHD (Figure 1). The catalytic subunit SDHA is the largest subunit and generates FADH2 by oxidizing succinate to fumarate as part of the TCA cycle. SDHB contains three iron–sulfur clusters that accept the electrons from FADH2 and transfer them to SDHC and SDHD, which are embedded in the inner mitochondrial membrane and constitute the CII function within the ETC serving as the site for ubiquinone (Q) binding and reduction to ubiquinol (QH2) [2,3,4].

Pathogenic variants affecting the SDH/CII genes result in mitochondrial dysfunction. This can manifest either as primary mitochondrial diseases [5,6], or as susceptibility to tumorigenesis [5,6]. Congenital SDH/CII deficiencies are due to bi-allelic pathogenic variants in one of the SDH/CII genes, consistent with an autosomal recessive inheritance pattern, and result in childhood diseases including Leigh syndrome, cardiomyopathy, and infantile leukodystrophies [5,6]. Most often, congenital SDH/CII deficiencies are caused by pathogenic variants in *SDHA*, whereas genetic alterations in *SDHB*, *SDHD*, and *SDHAF1* are less frequent [5,6]. SDH/CII deficiencies account for approximately 2% of mitochondrial diseases diagnoses [5,7]. Tumorigenesis associated with SDH/CII dysfunction typically occurs when a germline mutation in one of the alleles is followed by a random mutation in the other wild-type allele over the lifetime of the individual, resulting in bi-allelic inactivation of SDH/CII in that cell, which evolves into a tumor through clonal expansion [5,6]. Pathogenic variants in *SDHB* and *SDHC* are associated with the development of pheochromocytomas (PCCs) and paragangliomas (PGLs) [5,6]. These tumors are usually benign catecholamine-secreting tumors that originate from the chromaffin cells of the adrenal medulla in the case of PCCs and from the extra-adrenal chromaffin tissue in the case of PGLs [5,6]. The association between SDH/CII defects and tumor development is not limited to PGLs and PCCs, but extends to other solid tissue tumors, including gastrointestinal stromal tumors (GISTs), pituitary adenomas, and renal cell carcinomas [5,6,7].

Undoubtedly, a detailed knowledge of the pathophysiology of SDH/CII deficiencies holds the key to discovering novel and more specific therapies. In addition, it may aid in the identification of adjuvant therapy in the treatment of diseases associated with SDH/CII dysfunction. For instance, prevalent neurodegenerative disorders, such as Alzheimer’s disease, Parkinson’s disease, and Huntington’s disease, exhibit a decrease in SDH/CII activity in brain tissue [8]. 

In this narrative review, we examine the impact of SDH/CII dysfunction on mitochondrial metabolic pathways. First, we review the direct consequences of SDH/CII deficiency in the TCA and the ETC. Subsequently, we provide an in-depth review of recent research focusing on the metabolic adaptations and vulnerabilities in SDH/CII deficient cells.

## 2. TCA Cycle Truncation in SDH/CII-Deficient Cells

**Figure 2:**
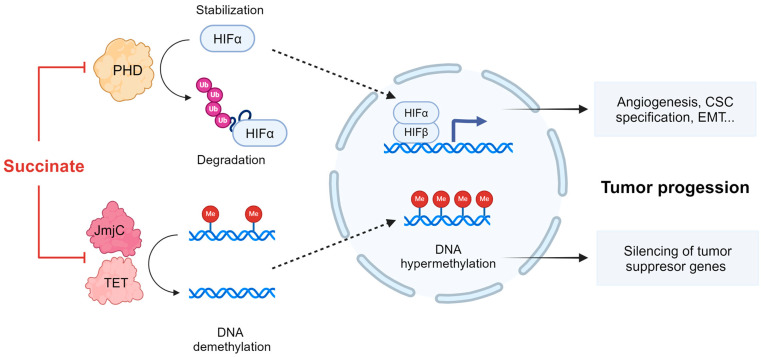
Consequences of succinate accumulation in cells. Elevated succinate levels inhibit PHD, promoting HIF-1α stabilization and its translocation to the nucleus, and also inhibit JmjC and TET enzymes, leading to DNA hypermethylation. These effects orchestrate the cellular events that promote tumor progression. CSC, cancer stem cells; EMT, epithelial–mesenchymal transition; JmjC, jumonji-domain histone demethylases; PHD, prolyl-4-hydroxylase; TET, ten-eleven translocation methylcytosine dioxygenases.

The TCA cycle is a housekeeping metabolic pathway essential for the generation of energy and biosynthetic intermediates. Dysfunction of the SDH/CII complex secondary to SDHx mutations directly blocks the oxidative TCA cycle, impairing aspartate synthesis and NADH production [2]. In addition, by preventing the conversion of succinate to fumarate, these mutations lead to accumulation of succinate that is shuttled from the mitochondrial matrix to the cytoplasm. Succinate is considered an oncometabolite, acting in part through the aberrant activation of transcription factors and global epigenetic reprogramming [9]. Indeed, high steady-state levels of succinate in SDHx-related tumors lead to abnormal stabilization of the hypoxia-inducible transcription factors HIF1α and/or HIF2α and subsequent expression of hypoxia-inducible genes, even in normoxia [10,11,12,13]. The stabilization of HIFα is due to the failure of the hydroxylation of two specific proline residues in the oxygen-dependent degradation domains of HIFα [14]. Hydroxylation of HIFα leads to recognition of HIFα by a ubiquitin ligase that uses pVHL as its substrate recognition component, resulting in polyubiquitylation and destruction of HIFα by the proteasome [15]. HIFα hydroxylation is carried out by a family of prolyl-4-hydroxylase (PHD) enzymes, which belong to a family of dioxygenases that metabolize oxygen and use α-ketoglutarate (AKG) as cosubstrate. During the hydroxylation reaction, AKG undergoes oxidative decarboxylation to produce succinate. Succinate functions as a competitive inhibitor of PHDs, thus promoting stabilization of HIFα in SDHx-related tumors (Figure 2). HIF activates the transcription of thousands of genes that mediate angiogenesis, cancer stem cell specification, cell motility, epithelial–mesenchymal transition, extracellular matrix remodeling, glucose and lipid metabolism, immune evasion, invasion, and metastasis [15]. Thus, it is highly likely that HIF overexpression contributes to the tumorigenesis of *SDHx*-mutated cells.

In addition to PHDs, succinate accumulation competitively inhibits other AKG-dependent dioxygenases, including jumonji-domain histone demethylases (JmjC) and the ten-eleven translocation (TET) enzymes which promote DNA demethylation by oxidizing 5-methylcytosine [9,12] (Figure 2). Inhibition of JmjC and TET dioxygenases results in hypermethylation of promoter regions (CpG islands) and transcriptional gene silencing. A study demonstrated that in a large PGL/PCC cohort, the promoters of genes associated with neuroendocrine differentiation (*PNMT*) and epithelial–mesenchymal transition (*KRT19*) were hypermethylated, resulting in transcriptional silencing [11]. Consistent with these findings, KRT19 transcripts were downregulated in metastatic PGL/PCC tumors related to SDHB mutations compared to non-metastatic ones [16]. Notably, promoter hypermethylation of *P16*, a cell cycle regulator that acts as a tumor suppressor, was prevalent in SDH-mutated tumors and correlated with short disease-related survival [17]. Finally, Morin et al. recently demonstrated synergistic roles of TET repression and pseudohypoxia in acquiring metastatic traits, providing a rationale for targeting HIF2α and DNA methylation in SDH-associated malignant tumors [12].

## 3. The ETC and OXPHOS in SDH/CII-Deficient Cells

The impact of SDH/CII impairment on the ETC activity was largely explored by measuring the oxygen consumption rate (OCR) before and after the addition of inhibitors to measure the key parameters of mitochondrial respiration. Notably, basal respiration, maximal respiratory capacity, and ATP-coupled production are severely impaired in several *SDHx*-deficient cancer cell models, including *SDHB* knockout (KO) MDA231 human breast cancer cells [18], *SDHD* KO HEK293 human embryonic kidney cells [19], *SDHB* mutant UOK269 human kidney cancer cells [20,21], *Sdha* KO and *Sdhb* KO RAW 264.7 mouse macrophage cells [22], and *Sdhb* KO mouse epithelial kidney cells [23]. This dramatic alteration in the respiratory parameters was not expected in the presence of an isolated SDH/CII defect. Consequently, several of the aforementioned studies further investigated the molecular mechanisms underlying these severe respiratory alterations and found a significant decrease in CI activity in SDH-deficient cell lines [18,20,23,24], whereas CIII, CIV, and CV remained largely unchanged [18,24]. Notably, decreased CI activity correlated with decreased protein expression of CI subunits, specifically the core subunits NDUFV1, NDUFS1, NDUFS8, MT-ND5, and MT-ND6, as well as the accessory subunits NDUFS4, NDUFS5, NDUFS6, NDUFA7, NDUFA9, NDUFA10, NDUFB3, NDUFB8, and NDUFB9 [18,20,23,24]. Regarding the effect of loss-of-function mutations in the catalytic subunit SDHA on CI activity, some data indicate that, in contrast to tumorigenic *SDHA* variants, neurodegenerative *SDHA* variants have either no effect or a very subtle effect on CI activity [24,25,26,27,28]. 

**Figure 3:**
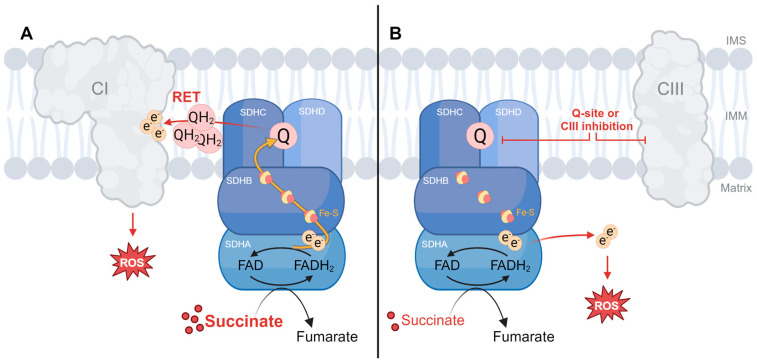
Mechanisms for ROS generation by SDH/CII. (**A**) Ubiquinol (QH2) overproduction by SDH/CII under high succinate levels is responsible for ROS generation in CI via reverse electron transport (RET). (**B**) ROS can be generated directly at the FAD site in SDHA under low succinate levels, provided that electron transport at the Q site or CIII is blocked. Fe-S, iron–sulfur clusters; IMM, inner membrane; and IMS, intermembrane space.

The ETC complexes CI and CIII, especially CI, are the major sites of mitochondrial ROS production under normal and stress conditions. The primary ROS generated by the ETC is superoxide O^2−^, which is easily converted to other ROS species, namely hydrogen peroxide (H_2_O_2_) and hydroxyl radical (OH). Excessive ROS levels lead to oxidative modifications and biomolecular damage, altering lipid/protein/DNA structure and functions. There is evidence that CII can also contribute to ROS production under certain circumstances. Firstly, CII may indirectly contribute to ROS generation via reverse electron transport (RET). This occurs at high succinate concentrations that allow CII to overproduce QH2. Electrons from QH2 are forced back to the FMN of the CI, leading to superoxide production as a result of the reaction between O_2_ and reduced FMN [29,30]. Secondly, ROS can be generated at the FAD site of SDHA when succinate is at low concentrations and the ETC is hindered at the Q site of SDH/CII or downstream (e.g., at CIII) [31,32,33,34]. These mechanisms of ROS production were demonstrated using competitive inhibitors and Q site inhibitors. The use of inhibitors such as 3-nitropropionic acid (3-NPA), malonate (MA), or methyl malonate (MMA), which compete with succinate for binding to the active site and thus prevent FAD reduction, attenuated ROS production [31]. In contrast, Q site inhibitors such as atpenin A5 (AA5), thenoyltrifluoroacetone (TTFA), diazoxide [34], and MitoVES [32], as well as the CIII inhibitors stigmatellin [34] and myxothiazole [33], induced ROS production. It is worth noting that ROS production at the FAD site of SDHA was shown to be inhibited when the concentration of succinate is high/saturating [32,33,34]. Finally, ROS production at the Q site of CII was also described, although this appears to be a rare mechanism in mammals [35]. Figure 3 summarizes the sites and conditions under which ROS generation by SDH/CII was documented.

In addition to the effects of SDH/CII inhibitors on ROS production, the effects of the genetic ablation of SDH/CII subunits on ROS production were explored. Silencing of SDHA in 143B human osteosarcoma cells did not alter normoxic ROS production [31]. In contrast, silencing of SDHB increased normoxic ROS production in both 143B human osteosarcoma cells and Hep3B human liver cancer cells [31]. These results are consistent with the uncoupled SDHA subunit being catalytically active and capable of generating ROS. However, they seem contradictory to a study using recombinant SDHA and SDHB proteins, which showed that unbound SDHA had minimal catalytic activity, whereas SDHA catalytic activity was maximal when bound to SDHB. These observations suggest that early assembly intermediates containing only the SDHA and SDHB subunits have uncoupled catalytic activity in cells [36].

SDH/CII can work in reverse when O_2_ is limited, removing electrons from the QH2 pool to reduce fumarate to succinate. Remarkably, a recent work showed that the reduction in fumarate by SDH/CII is critical for maintaining the activities of CI and dihydroorotate dehydrogenase (DHODH), as well as for proliferation in cells unable to use O_2_ as a terminal electron acceptor [37]. CI is the major site controlling NADH oxidation, while DHODH, a key enzyme of the de novo pyrimidine synthesis pathway, catalyzes the oxidation of dihydroorotate. In both cases, these oxidation reactions are coupled to the reduction in Q to QH2, providing electrons to CIII. The study shows that inhibition of electron flux downstream of QH2 through genetic ablation of key subunits of complex III (UQCRC2) or complex IV (COX4) in 143B human osteosarcoma cells did not affect DHODH activity and only modestly decreased CI activity. This unexpected result was coupled with an increase in SDH/CII reverse activity and fumarate reduction, as assessed by isotopic labeling with glutamine. The essential role of QH2 accumulation in reversing SDH/CII activity was demonstrated by the suppression of fumarate reduction in UQCRC2 or *COX4* KO cells upon expression of AOX, which converts QH2 to Q. This finding is in line with the higher reduction potential of Q as compared to fumarate. Consistent with the results obtained by genetic inactivation of CIII, pharmacological inhibition of CIII using antimycin resulted in increased fumarate reduction in primary dermal fibroblasts and in a panel of human cancer cell lines including 143B, SW1353, U87, DLD1, and HCT116 [37]. Unequivocal confirmation of the crucial role of fumarate reduction in maintaining CI and DHODH activities came when ablation of *SDHB* rendered cells unable to maintain these activities under antimycin treatment. The study finally moves to in vivo experiments and illustrates that hypoxia causes fumarate reduction in mouse tissues through in vivo isotopic tracing [37].

## 4. Metabolic Adaptations and Vulnerabilities Imposed by Deficiencies in SDH/CII

**Figure 4:**
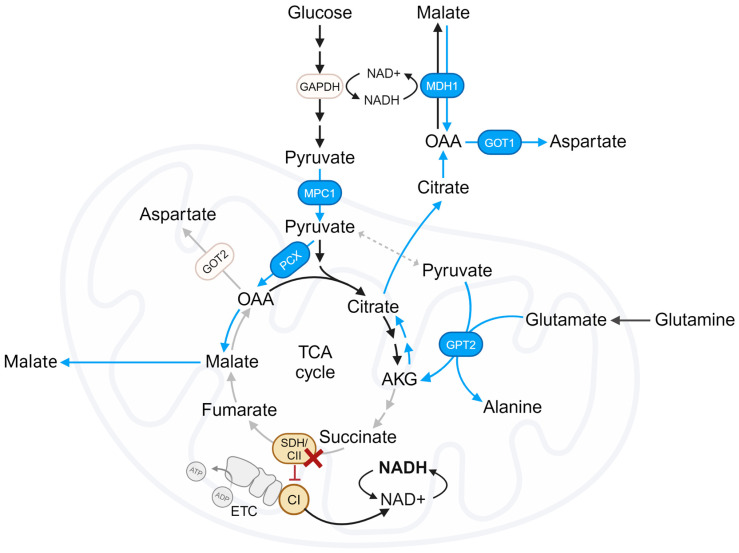
Metabolic adaptations imposed by SDH/CII deficiencies. Schematic detailing how aspartate is synthesized in SDH/CII-deficient cells when CI is also impaired. Forward reactions are indicated by black/gray arrows and reverse reactions are indicated by blue arrows. AKG, α-ketoglutarate; ETC, electron transport chain; GAPDH, glyceraldehyde-3-phosphate dehydrogenase; GOT1/2, glutamate-oxaloacetate transaminase 1/2; MDH1/2, malate dehydrogenase 1/2; MPC1, mitochondrial pyruvate carrier 1; OAA, oxaloacetate; and PCX, pyruvate carboxylase.

The TCA cycle produces reducing equivalents NADH and FADH2, essential for transferring electrons to the ETC, and is required to support continuous production of aspartate. Aspartate is a critical amino acid for purine and pyrimidine synthesis and cell proliferation. Recently, a few studies published in high-impact journals present evidence on how cells deficient in SDH/CII, which have a disrupted TCA cycle, adapt their metabolism to synthesize aspartate and sustain cell growth. The key findings were grouped into sections and are discussed below. Figure 4 graphically represents the major metabolic pathways used by SDH/CII-deficient cells to survive.

### 4.1. Reliance on Pyruvate Carboxylation

Two pioneering studies from 2015 showed that SDH/CII loss leads to a dependence on pyruvate carboxylation (PC) for aspartate synthesis and proliferation [23,38]. These findings are based on metabolic flux analysis in immortalized *Sdhb* KO mouse cell lines from different lineages: kidney cells, chromaffin cells (imCC), and adrenal fibroblasts (MAF). *Sdhb* KO cell lines preferentially synthesize aspartate from glucose by carboxylation of pyruvate to oxaloacetate (OAA) and subsequent production of aspartate. In contrast, the primary source of aspartate in wild-type cells is glutamine via the oxidative TCA cycle. As expected, the incorporation of glutamine carbons into aspartate via the oxidative TCA cycle was undetectable in *Sdhb* KO cells, consistent with their truncated TCA cycle [23,38]. Additionally, these studies claim that glutamine contributes minimally to aspartate production through the reductive TCA cycle, which contrasts with more recent findings summarized in the following sections. In this regard, treatment with a glutaminase inhibitor reduced OAA levels in wild-type cells but not in *Sdhb* KO cells, further supporting the notion of a low contribution of glutamate as a source of aspartate in *Sdhb* KO cells [38]. In summary, these landmark studies seem to demonstrate that glucose, via the PC pathway, is the major contributor of aspartate production in SDH/CII-deficient cells. Despite the activation of the PC pathway, steady-state levels of aspartate were significantly decreased in *Sdhb* KO cells and were further reduced upon pyruvate deprivation. Similarly, analysis of amino acid concentrations in SDH-mutated PCC/PGL tumors showed that aspartate concentrations were the most affected [38]. The dependence of *Sdhb* KO cells on pyruvate carboxylase (PCX) activity for survival was confirmed by the observation that silencing PCX resulted in decreased proliferation in these cells, which could be counteracted by the addition of aspartate [23,38]. The role of PCX in *Sdhb* KO cell survival from in vitro experiments was further supported by the detection of elevated PCX in SDH-mutated PCC/PGL tumors [23].

### 4.2. Contribution of the Reductive TCA Cycle

Reductive TCA cycle metabolism was previously identified as a mechanism for macromolecular synthesis in cells with defective mitochondria, including growing cancer cells [39]. Accruing evidence suggests that glutamine is an important contributor of carbon to the TCA cycle for biomass synthesis through reductive carboxylation (RC), which is a process by which glutamine-derived AKG is reduced in the non-canonical reverse reaction, to form citrate [40,41,42]. Glutamine is converted to AKG in two steps: first, into glutamate via glutaminolysis, and then into AKG through pathways involving glutamate dehydrogenase or various transaminases, such as glutamate–pyruvate transaminase, glutamate-oxaloacetate transaminase, and phosphoserine transaminase [40,43]. 

Recently, Ricci et al. demonstrated that the reductive carboxylation (RC) pathway, initiated by the glutamate pyruvate transaminase (GPT) isoform 2, plays a crucial role in aspartate production and the proliferation of SDH/CII-deficient cells [44]. The transaminase GPT2 is localized to mitochondria and catalyzes the reversible transamination between glutamate and pyruvate, yielding AKG and alanine [45,46]. Initially, the study by Ricci et al. highlights increased Gpt2 activity in *Sdhb* KO mouse kidney cell lines as evidenced by increased incorporation of glutamine nitrogens into alanine and elevated steady-state alanine levels compared to their controls. The dependence of *Sdhb* KO cells on Gpt2 was confirmed by observing that knockdown of Gpt2 expression selectively reduced the proliferation of *Sdhb* KO cells while sparing control cells. Notably, the RC pathway initiated by GPT2 was also identified as essential for the survival of other cell models with defective mitochondria [47,48,49,50]. To broaden the translational implications of these findings, analysis of publicly available human transcriptomic data reveal that decreased SDHx expression or activity in tumors driven by oncogenic *SDHx* mutations correlated with upregulation of GPT2 mRNA levels [44]. In addition, results from RNA interference screens in the Project Achilles database revealed a significant correlation between depletion of shRNAs targeting GPT2, indicating cell sensitivity to GPT2 loss and SDHB hypoexpression [44]. These results obtained from the analysis of public databases should be confirmed through experimental approaches both in vitro and in vivo.

As mentioned above, the conversion of pyruvate to alanine by GPT2 is coupled to the conversion of glutamate to AKG. Significantly, the decrease in AKG production observed in *Sdhb* KO cells upon Gpt2 knockdown is responsible for their proliferative deficiency, as supplementation with AKG significantly rescued proliferation [44]. Tracing the fate of glutamine carbons revealed Gpt2-dependent AKG generation drove the reverse flow of the TCA cycle, thus bypassing the oxidative TCA cycle truncation imposed by SDH inactivation [44]. AKG is converted into citrate, which is exported to the cytosol and subsequently converted into OAA by ATP-citrate lyase (ACLY) [48]. This pool of OAA fuels the activity of cytosolic malate dehydrogenase 1 (MDH1), essential to regenerate NAD+ consumed by glyceraldehyde-3-phosphate dehydrogenase (GAPDH), a pivotal enzyme in the glycolysis pathway [44,47]. *Sdhb* KO cells showed normal steady-state NAD+ levels; however, as expected, Gpt2 silencing in *Sdhb* KO cells led to a decrease in the NAD+/NADH ratio, accumulation of glycolytic intermediates upstream of the GAPDH-catalyzed step, and a decrease in ATP levels [44]. Supplementation with NAD+ or aspartate, which acts as a source of OAA to fuel MDH1-dependent activity in cells with mitochondrial dysfunction, significantly rescued the proliferative fitness of SDH-deficient cells impaired in response to the suppression of Gpt2 expression. Furthermore, pharmacological inhibition of NAD+ biosynthesis with FK866 limited ATP production and proliferation in *Sdhb* KO cells. Interestingly, the combination of FK866 with the SDH/CII inhibitor α-TOS, but not separately, had antitumor effects on cancer cells transplanted into mice [44]. 

### 4.3. The Role of CII-Low

The subunits of SDH/CII are noted for their ability to integrate into alternative species, potentially influencing signaling pathways [51]. In mammalian cells, SDHA is the only subunit that is a stable part of a species of approximately 100 kDa that is termed CII-low because it has a lower molecular weight than SDH/CII. CII-low represents a heterogeneous mixture of species that, in addition to the catalytic subunit SDHA, contain the assembly factors SDHAF2 and SDHAF4 [18,51,52,53]. The predominant species is likely the stable SDHA-SDHAF2 complex. Notably, CII-low accumulates in SDHB-deficient PGL as well as in SDHB- and SDHC-deficient cell lines and, as shown by a single study by Bezawork-Geleta et al., partially contributes to the metabolic adaptations observed in SDH-deficient cells [18]. This study compares metabolic adaptations between the *SDHB* KO human breast adenocarcinoma cell line MDA231 and *SDHB* KO MDA231 cells in which SDHA was silenced, resulting in the absence of CII-low. The depletion of CII-low in *SDHB* KO cells resulted in increased incorporation of glucose and glutamine carbons into aspartate via PC and RC pathways, respectively. Intriguingly, this depletion also facilitated the conversion of glucose- and glutamine-derived succinate to fumarate, and ultimately to aspartate, although to a limited extent by mechanisms that remain to be understood [18]. Aspartate plays a critical role in de novo pyrimidine synthesis and cell proliferation. Consequently, the depletion of CII-low partially reversed the downregulation of the de novo pyrimidine synthesis pathway and the S-phase arrest observed in *SDHB* KO cells. This finding seems to contradict the link between the accumulation of CII-low and tumorigenesis. However, when *SDHB* KO MDA231 cells were transplanted into mice, they were able to form tumors, whereas *SDHB* KO MDA 231 cells with silenced CII-low failed to do so. Furthermore, consistent with the in vitro data, examination of PGL tissue from patients with elevated CII-low levels showed reduced levels of CAD, a multifunctional protein that participates in the initial steps of the de novo pyrimidine synthesis pathway [18]. The authors propose that CII-low serves as a sensor for bioenergetic stress within tumors, enabling them to regulate metabolism by suppressing high-energy-demand cellular processes, such as the synthesis of aspartate, a vital component in pyrimidine nucleotide synthesis [18].

### 4.4. The Benefits of CI Inhibition

A recent study by Hart et al. demonstrates that the absence of CI in SDH/CII-deficient cells, described in Section 3, serves as an adaptive mechanism. The authors show that the loss of CI occurs gradually over passages in *SDHB* KO cells, and that this loss is beneficial to CII-deficient cells by increasing their production of aspartate, their proliferation rate, and their capacity for tumor growth [20]. CI, as is the case with CII, is an entry point into the ETC. CI, also known as NADH:ubiquinone, catalyzes the transfer of two electrons from NADH to ubiquinone in a reaction that is associated with proton translocation across the membrane [54]. The study elegantly demonstrates how the reduction in CI activity enables essential alternative mitochondrial and cytosolic pathways of aspartate synthesis in SDH/CII-deficient cells. Furthermore, the essential role of CI reduction in the health of SDH-deficient cells is highlighted by the deleterious effect of overexpressing *Saccharomyces cerevisiae* NDI1 [55], a rotenone-insensitive CI analog that can restore NADH oxidation and ETC activity in mammalian cells when CI is impaired, in *SDHB*-deficient cells.

To avoid the adaptive decrease in CI and thus explore the impact of CI activity in the context of CII deficiency, this study uses pharmacological inhibitors for short periods of time or *SDHB* KO cells at early passages. The work begins with the observation that the Q-site inhibitor AA5 blocked the proliferation of 143B human osteosarcoma cells, and that the degree of proliferation inhibition correlated with aspartate steady-state levels. Consistent with previous data that low aspartate levels in SDH-deficient cells mediate proliferation defects, proliferation of AA5-treated cells was restored by approaches that increase intracellular aspartate levels, including aspartate supplementation and the expression of SLC1A3 [56], a transporter involved in aspartate import across the plasma membrane in glial cells. As expected, AA5 treatment increased the mitochondrial NAD+/NADH ratio. This was attributed to decreased mitochondrial NADH levels resulting from TCA cycle blockade, coupled with simultaneous NADH oxidation by active mitochondrial CI. However, the cytosolic NAD+/NADH ratio remained unchanged. This study introduces the increased mitochondrial NAD+/NADH ratio as a novel mechanism for reducing aspartate levels and impairing proliferation in AA5-treated cells, as co-inhibition with the CI inhibitor rotenone, which restores the mitochondrial NAD+/NADH ratio by reducing NAD+, rescues the phenotype. Ectopic expression of *Lactobacillus brevis* NADH oxidase (LbNOX) in the mitochondria [57] prevented the beneficial effects of rotenone treatment on aspartate levels and cell proliferation in AA5-treated cells. This underscores the importance of NAD+ depletion for the advantages of CI inhibition. 

Next, the work explored the source of the increased aspartate in SDH-deficient cells upon CI inhibition by performing glucose and glutamine isotope incorporation experiments in wild-type 143B cells and low-passage *SDHB* KO cells. Consistent with previous research [23,38], glutamine served as the major source of aspartate through oxidative TCA cycle metabolism in wild-type cells, whereas glucose had a minor role in contributing to aspartate. In *SDHB* KO cells, glucose was the primary source of aspartate via PC, with rotenone treatment further increasing the flow of glucose carbons into aspartate. Glutamine contributed to aspartate production in these cells via the RC pathway, which was enhanced in the presence of rotenone; whereas, as expected, glutamine-derived aspartate production via oxidative TCA was abolished. Consistent with these findings, AA5 treatment significantly reduced aspartate production and proliferation in *PCX* KO cells to a much greater extent than in wild-type cells. However, rotenone treatment still provided a proliferative benefit to AA5-treated *PCX* KO cells. This residual proliferative benefit was attributed to the RC pathway, as its inhibition using an inhibitor of ACLY reversed the advantage conferred by rotenone.

Both the PC and RC pathways involve reactions that can occur either in the cytosol or in the mitochondria. This study elucidates the specific cytosolic and mitochondrial reactions underlying the beneficial effects of CI loss in SDH-deficient cells. First, the dependence on mitochondrial pyruvate availability was investigated by generating *MPC1* KO cells, since MPC1 is a critical subunit of the mitochondrial pyruvate carrier [58]. In these KO cells, proliferation and aspartate production were completely ablated by SDH impairment and were no longer enhanced by CI co-inhibition. This finding indicates that mitochondrial pyruvate drives the rotenone-mediated increase in PC and RC pathways in SDH-deficient cells. This was expected since mitochondrial pyruvate serves as a substrate for the PC pathway and is also required for the GPT2 reaction, which, as previously described [44], is the dominant pathway for glutamate conversion to AKG in SDH-deficient cells, thereby fueling aspartate production via the RC pathway. The enzyme responsible for aspartate production is the transaminase GOT, which converts glutamate and OAA to aspartate and AKG [59]. GOT exists in cytosolic and mitochondrial forms, GOT1 and GOT2, respectively. The knockout of GOT isoforms revealed that GOT1, but not GOT2, is crucial for aspartate production in SDH-impaired cells under CI inhibition. Notably, CI inhibition still increased malate levels in SDH-impaired *GOT1* KO cells, suggesting that the mitochondrial redox changes from CI inhibition drive the biochemical pathways required for alternative aspartate synthesis in these cells (PC and RC), but that cytosolic GOT1 is required for terminal aspartate generation. Similarly, GOT1 was shown to drive aspartate synthesis in cells with ETC inhibition at CI and CIII [60]. Since CI inhibition still increased malate levels in SDH-impaired *GOT1* KO cells, they next investigated the role of cytosolic MDH1 in the benefits of CI inhibition in SDH-deficient cells. Cytosolic MDH1 catalyzes the reduction in OAA to malate using the oxidation of NADH to NAD+ in the forward malate–aspartate shuttle [61]. Similar to *GOT1* KO, *MDH1* KO prevented CI inhibition from restoring aspartate levels in SDH-impaired cells, indicating that MDH1 acts in the reverse direction. Thus, in SDH-deficient cells, cytosolic malate would be oxidized to OAA by MDH1 using the reduction in NAD+ to NADH prior to transamination to aspartate by GOT1 (Figure 4). This is in apparent contradiction to previously mentioned studies [44,47] that argue that the forward MDH1 reaction, which converts OAA into malate, is essential to regenerate NAD+ consumed by glyceraldehyde-3-phosphate dehydrogenase (GAPDH), and therefore, to maintain the glycolysis pathway in cells with mitochondrial dysfunction. For aspartate produced from pyruvate-derived OAA, the dispensability of mitochondrial GOT2 for aspartate production upon CI inhibition in CII-deficient cells, together with the different NAD+/NADH ratios in the cytosol and mitochondria, supports the reverse malate–aspartate shuttle. In this reverse shuttle, mitochondrial MDH2 reduces pyruvate-derived OAA to malate, which is then exported to the cytosol and oxidized to OAA by MDH1 before being transaminated to aspartate by GOT1. Notably, the reverse malate–aspartate shuttle was also observed in other respiration-deficient cell models, including those with CIII inhibition [62]. Despite these advances in understanding the pathways controlling aspartate production and proliferation in SDH-deficient cells, further research is needed to elucidate these coordinated mitochondrial and cytosolic reactions and to clarify the apparent contradictions between different studies.

## 5. Conclusions and Future Directions

Undoubtedly, the most effective therapies can only result from advances in the understanding of the molecular processes underlying disease. In the first place, this review suggests that HIF-1 may be a promising therapeutic target for cancers associated with CII/SDH deficiency. In this regard, it is important to highlight that several phase II clinical trials are investigating HIF-1 inhibitors (such as LBH589, SCH66336, echinomycin, and 2ME2) for various cancers, including ovarian, breast, and colorectal cancers. However, these inhibitors are yet to be used to treat cancers associated with SDH/CII subunit mutations, such as PGLs and PCCs [63,64]. What also is learned from this review is that the most exciting emerging data focus on the metabolic adaptations imposed by SDH/CII deficiency and the specific cellular vulnerabilities. This review identifies several promising targets in SDH/CII-deficient cells, including PCX, GPT2, MDH1, and MPC1, among others, that urgently need to be tested. These targets offer dual intervention strategies: improving metabolic homeostasis or inducing cell death, depending on the therapeutic goal.

Despite the progress in understanding the pathophysiology of SDH/CII-associated diseases summarized in this review, significant knowledge gaps remain. This is somewhat surprising given that SDH/CII is an enzyme that was characterized for a long time. For example, it is essential to further elucidate the pathways used by SDH/CII-deficient cells in both the cytosol and mitochondria to produce aspartate and support proliferation. In addition, it is important to resolve the apparent contradictions between different studies. Additionally, as mentioned in Section 3, some data suggest that loss-of-function mutations in the catalytic subunit SDHA linked to tumorigenesis are associated with changes in CI activity, whereas neurodegenerative SDHA variants have minimal or no effect on CI activity. This has important implications, indicating that therapies must be personalized based on the specific patient mutation. However, these findings are extremely preliminary, being studied in only a few different SDHA mutations. Furthermore, the role of free SDHA (CII low) in the metabolic adaptations of SDH/CII-deficient cells is yet to be thoroughly explored. Most current studies investigating the impact of SDHA on the metabolism of SDH/CII-deficient cells use SDHA pharmacological inhibitors or SDHB-deficient cells in which SDHA was silenced with shRNAs. Novel genetic approaches need to be developed to explore the role of free SDHA in diseases associated with SDH/CII deficiencies. Additionally, it would be valuable to explore the molecular mechanisms by which structural and functional loss of SDH/CII inhibits CI biogenesis. The pathways governed by SDH/CII that affect CI activity remain unknown and understanding them is essential for potential interventions. A recent study underscores the importance of fumarate reduction in maintaining CI and dihydroorotate dehydrogenase (DHODH) activities under low oxygen conditions [37]. Given that solid tumors often exist in low-oxygen microenvironments, this observation is particularly relevant. The decreased fumarate levels in SDH/CII-deficient cells might explain the reduced CI activity.

In conclusion, it is imperative to advance research in this field, which, despite being well-established, progressed relatively slowly compared to others. Advancing our knowledge in this area will enable the development of personalized treatments for patients with diseases related to SDH/CII functions.
